# Intravenous Cone-Beam CT for Follow-up of Intracranial Aneurysms Treated with the Woven EndoBridge Device

**DOI:** 10.1007/s00062-025-01576-7

**Published:** 2025-10-21

**Authors:** Yosuke Tajima, Masaaki Kubota, Hajime Yokota, Jun Koizumi, Katsuya Hayashi, Yoshinori Higuchi

**Affiliations:** 1 Department of Neurological Surgery, Chiba University Hospital, 1-8-1 Inohana, 260-8677 Chiba, Japan; 2 Diagnostic Radiology and Radiation Oncology, Graduate School of Medicine, Chiba University, Chiba, Japan; 3 Department of Radiology, Chiba University Hospital, Chiba, Japan

**Keywords:** Digital subtraction angiography, Intracranial aneurysm, Intravenous cone-beam computed tomography, Woven EndoBridge device

## Abstract

**Purpose:**

The Woven EndoBridge (WEB) device is an established endovascular treatment method for wide-neck intracranial aneurysms. Digital subtraction angiography (DSA) is the reference standard for posttreatment evaluation; however, its invasive nature limits its repeated use. This study aimed to compare the diagnostic accuracy and reproducibility of intravenous cone-beam computed tomography (IVCBCT) and DSA in the follow-up of aneurysms treated with the WEB device.

**Methods:**

This prospective single-center study included 16 patients with unruptured intracranial aneurysms who were treated with the WEB device. All patients underwent both DSA and IVCBCT at the 6‑month follow-up, performed on the same day. Aneurysm occlusion status was independently evaluated by two experienced neuroradiologists using the 8‑point modified Bicêtre Occlusion Scale Score (BOSS). Intermodality and interobserver agreement were assessed using Cohen’s kappa statistics.

**Results:**

Complete agreement between IVCBCT and DSA was observed in all 16 cases (κ = 1.0 for both raters). Interobserver agreement for IVCBCT grading was also excellent (κ = 0.86). The BOSS distribution for IVCBCT was as follows: grade 0 or 0’ in eight cases (50.0%), grade 1 in five cases (31.3%), grade 2 in two cases (12.5%), and grade 3 in one case (6.2%). IVCBCT demonstrated consistent diagnostic performance across various occlusion grades.

**Conclusion:**

IVCBCT offers a reliable, noninvasive alternative to DSA for the follow-up of WEB-treated aneurysms, providing excellent diagnostic concordance and reproducibility.

## Introduction

The Woven EndoBridge (WEB; MicroVention, Aliso Viejo, CA, USA) is a well-established endovascular device designed to treat wide-neck intracranial aneurysms—typically defined as a dome-to-neck ratio < 2 or a neck width ≥ 4 mm—by promoting intra-aneurysmal thrombosis [1, 2]. Its safety and efficacy have been demonstrated in multiple clinical studies [3, 4]. As with other endovascular therapies, imaging follow-up is essential to detect potential aneurysm recurrence. Digital subtraction angiography (DSA) remains the reference standard for posttreatment evaluation; however, it is invasive and may lead to neurological complications. Nam et al. reported an overall complication rate of 5% following DSA procedures, with neurological complications occurring in approximately 1% of cases [5]. Recently, intravenous cone-beam computed tomography (IVCBCT) has emerged as a promising non-invasive alternative, offering spatial resolution comparable to that of intra-arterial CBCT, with lower risk and simultaneous opacification of all intracranial vascular territories [6, 7]. However, its performance relative to that of DSA has not been clearly determined for the patients treated with the WEB device. In this single-center prospective study, the diagnostic accuracy of IVCBCT was compared with that of DSA for evaluating aneurysm occlusion after WEB device implantation.

## Materials and Methods

### Population

This single-center prospective study was approved by our local ethics committee (Institutional Board No. 10305), and informed consent was obtained from all participants. Sixteen patients treated with the WEB device at our hospital between March 2023 and October 2024 and followed up for 6 months were retrospectively analyzed. Patients who did not undergo DSA follow-up at 6 months were excluded from the study. DSA and IVCBCT were performed on the same day for all included patients to ensure consistency in evaluation.

### Imaging Technique for IVCBCT

An 18-gauge cannula was inserted into the median cubital vein, and 95 mL of iopamidol (370 mg/mL, Hikari Pharmaceutical Co., Ltd., Tokyo, Japan), followed by a 30 mL saline chase, was injected at 5 mL/s using a powered injector. Seventeen seconds after the injection, CBCT was performed to acquire IVCBCT images. IVCBCT data were acquired using interventional C‑arm cone-beam CT systems Allura Xper FD20/20 and XperCT (Philips Healthcare, Best, the Netherlands), using the following parameters: Allura Xper 20; acquisition time, 20 s; tube voltage, 80 kV; projection matrix, 1024 × 1024; rotation angle, 210°; total frames, 622; dose, 0.9 μGy/pulse.

Raw projection data were reconstructed using a filtered back-projection algorithm based on the Feldkamp-Davis-Kress method, yielding an isotropic voxel size of 0.2 mm^3^ [8]. Reconstruction was performed on the XperCT workstation (Philips Healthcare), with the following parameters: a medium-sharp convolution kernel (Hounsfield unit-based), edge-enhancement filters, and a 512^3^ matrix size.

Multiplanar reformatted images were generated in axial, sagittal, and coronal planes. In addition, maximum intensity projection and volume-rendered images were created with default preset parameters. All reconstructed datasets were exported to the institutional picture archiving and communication system (PACS; Synapse, Fujifilm Medical, Tokyo, Japan) for diagnostic interpretation. During image review, readers were allowed to perform real-time multiplanar reformatting, window-level adjustment, zooming, and image rotation using PACS-native tools to ensure optimal visualization of aneurysm occlusion and intrasaccular contrast distribution.

### Imaging Technique for DSA

DSA was performed using a biplane angiographic system (Allura Clarity FD 20/15, Philips). Using transarterial catheterization, we performed selective injections in the common carotid artery or vertebral artery via a 4F catheter according to the location of the aneurysm. Frontal and lateral views were obtained with an 8 mL bolus of iopamidol (300 mg/mL) injected at 4–6 mL/s.

### Angiographic Evaluation: Imaging Diagnostic Quality and Performance

Two neuroradiologists with more than 15 years of experience independently evaluated both IVCBCT and DSA images in random order, using a standardized workstation (Philips Healthcare). Readers were permitted to freely adjust windowing and perform multiplanar reformats, but the same preset protocol was used as a default. Pretreatment DSA images were available to assist in anatomical orientation. Each reader assessed the IVCBCT and DSA images in separate sessions at least one month apart to minimize memory bias. The readers were blinded to the results of the other modality and to their previous assessments.

Aneurysm occlusion was assessed using identical imaging parameters for both modalities, based on the Bicêtre Occlusion Scale Score (BOSS). According to the original BOSS, occlusion is defined as follows: BOSS 0 = no residual flow; BOSS 0’ = proximal recess opacification; BOSS 1 = residual flow within the device with complete neck sealing; BOSS 2 = neck remnant; BOSS 3 = aneurysm remnant; and BOSS 1 + 3 = flow within both the device and sac without neck sealing [9].

Furthermore, a modified BOSS grading system proposed by Caroff et al. was used, subdividing BOSS 1 into three categories based on the extent of intradevice opacification: BOSS 1^33%^ (1–33%), BOSS 1^66%^ (34–66%), and BOSS 1^100%^ (67–100%) [10]. Although not yet standardized, this subclassification offers finer granularity in assessing occlusion following WEB treatment and has been adopted in our institutional practice. This finer grading system is aimed at better reflecting the diversity of residual flow patterns specific to WEB-treated aneurysms.

### Statistical Analysis

Quantitative variables are reported as extremes, means ± SD, and medians (interquartile range), while qualitative variables are reported as numbers and percentages. The Cohen’s kappa coefficient κ was used to evaluate intermodality agreement between IVCBCT and DSA for each neuroradiologist and interobserver agreement for IVCBCT and DSA. The κ statistics were interpreted according to Landis and Koch [11].

## Results

Sixteen patients with unruptured intracranial aneurysms who were treated with the WEB device were included in this study. The mean age was 72 ± 8 years (range, 56–85 years), with 14 women (87.5%) and 2 men (12.5%). All patients underwent IVCBCT and DSA between 6 and 12 months post-treatment (mean follow-up, 7.4 ± 2.6 months). The aneurysm locations included the anterior communicating artery (*n* = 5), middle cerebral artery bifurcation (*n* = 5), terminal basilar artery (*n* = 4), and internal carotid artery (*n* = 2). The mean aneurysm dome size was 7.1 ± 2.0 mm, and the mean neck width was 4.5 ± 1.2 mm. All aneurysms were treated with the WEB device without adjunctive devices.

Aneurysm occlusion was assessed using the modified BOSS, which incorporates intradevice opacification subgrades (BOSS 1^33%^, 1^66%^, 1^100%^). Complete concordance between IVCBCT and DSA was observed in all 16 cases for both raters (100% agreement). The intermodality agreement between IVCBCT and DSA for each reader yielded a Cohen’s kappa coefficient of 1.0, indicating perfect agreement. Regarding interobserver agreement for IVCBCT-based assessments, a high level of consistency was achieved with a Cohen’s kappa of 0.86 (95% CI: 0.68–1.0). Notably, the interobserver agreement for DSA-based BOSS was also substantial, with a Cohen’s kappa of 0.86. No significant discrepancies were observed between the two readers. A summary of the grading results for each modality and rater is provided in Table 1. The distribution of BOSS grades for IVCBCT was as follows: BOSS 0 or 0’ in eight cases (50.0%), BOSS 1^33%^ in two cases (12.5%), BOSS 1^66%^ in one case (6.3%), BOSS 1^100%^ in two cases (12.5%), BOSS 2 in two cases (12.5%), and BOSS 3 in one case (6.3%).

**Table 1:** Agreement between the IVCBCT and DSA evaluations for each rater.

Case	Rater A—DSA	Rater A—IVCBCT	A Match	Rater B—DSA	Rater B—IVCBCT	B Match
Case 1	BOSS 3	BOSS 3	Matched	BOSS 3	BOSS 3	Matched
Case 2	BOSS 1^66%^	BOSS 1^66%^	Matched	BOSS 1^66%^	BOSS 1^66%^	Matched
Case 3	BOSS 1^100%^	BOSS 1^100%^	Matched	BOSS 1^100%^	BOSS 1^100%^	Matched
Case 4	BOSS 1^100%^	BOSS 1^100%^	Matched	BOSS 1^100%^	BOSS 1^100%^	Matched
Case 5	BOSS 0’	BOSS 0’	Matched	BOSS 0’	BOSS 0’	Matched
Case 6	BOSS 0	BOSS 0	Matched	BOSS 0’	BOSS 0’	Matched
Case 7	BOSS 1^33%^	BOSS 1^33%^	Matched	BOSS 1^33%^	BOSS 1^33%^	Matched
Case 8	BOSS 2	BOSS 2	Matched	BOSS 2	BOSS 2	Matched
Case 9	BOSS 0	BOSS 0	Matched	BOSS 0	BOSS 0	Matched
Case 10	BOSS 1^33%^	BOSS 1^33%^	Matched	BOSS 1^33%^	BOSS 1^33%^	Matched
Case 11	BOSS 0	BOSS 0	Matched	BOSS 0	BOSS 0	Matched
Case 12	BOSS 0	BOSS 0	Matched	BOSS 0	BOSS 0	Matched
Case 13	BOSS 0	BOSS 0	Matched	BOSS 0	BOSS 0	Matched
Case 14	BOSS 2	BOSS 2	Matched	BOSS 2	BOSS 2	Matched
Case 15	BOSS 0	BOSS 0	Matched	BOSS 0	BOSS 0	Matched
Case 16	BOSS 0	BOSS 0	Matched	BOSS 0’	BOSS 0’	Matched

“Matched” indicates identical BOSS scores between modalities

*BOSS* bicêtre occlusion scale, *IVCBCT* intravenous cone beam computed tomography, *DSA* digital subtraction angiography

**Fig. 1:**
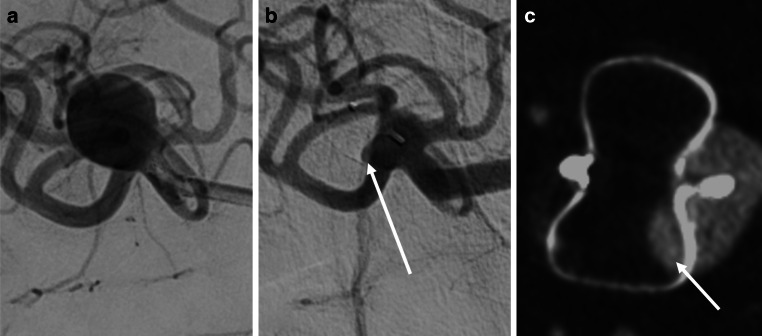
A representative case of a middle cerebral artery bifurcation aneurysm treated with the Woven EndoBridge (WEB) device. **a** Digital subtraction angiography (DSA) prior to WEB placement. (**b**) DSA and (**c**) intravenous cone-beam computed tomography (IVCBCT) performed at the 6‑month follow-up demonstrate a BOSS of 1^33%^ (minimal contrast opacification within the WEB device without neck remnants)

**Fig. 2:**
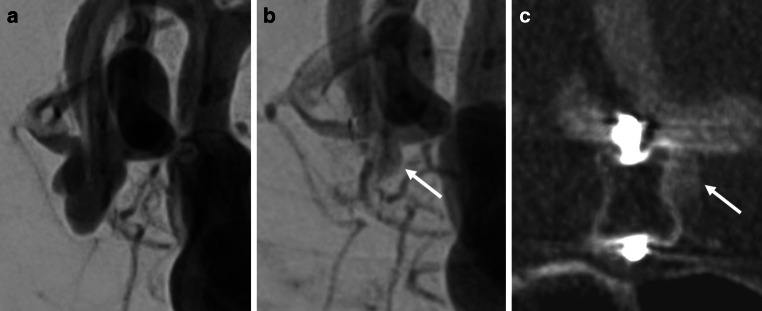
A representative case of an anterior communicating artery aneurysm treated using the Woven EndoBridge (WEB) device. **a** Digital subtraction angiography (DSA) prior to WEB placement. (**b**) DSA and (**c**) intravenous cone-beam computed tomography (IVCBCT) at the 6‑month follow-up showing an aneurysm remnant outside of the WEB device (white arrows) classified as BOSS 3

**Fig. 3:**
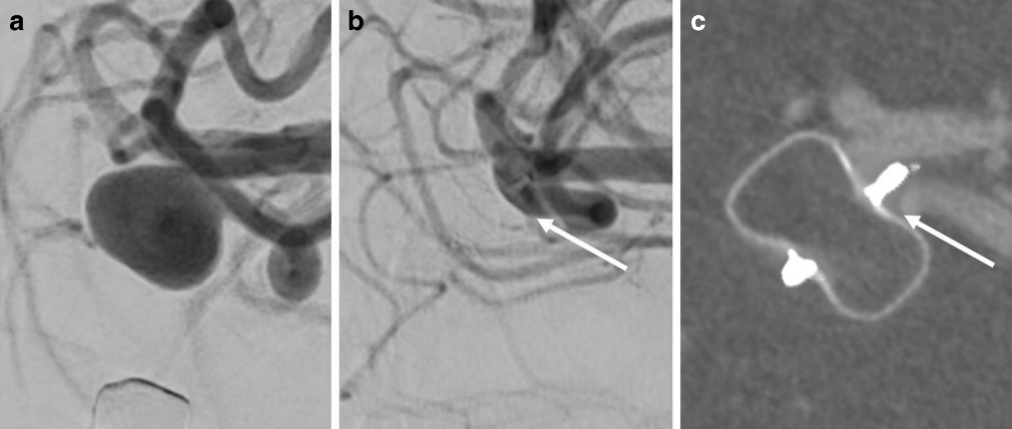
A representative case of a middle cerebral artery aneurysm treated with the Woven EndoBridge (WEB) device, illustrating a grading discrepancy between readers. (**a**) Digital subtraction angiography (DSA) and (**b**) intravenous cone-beam computed tomography (IVCBCT) at 6 months post-treatment both show subtle contrast opacification within the proximal recess of the device (white arrows). One reader classified the occlusion as BOSS 0 (complete occlusion), whereas the other classified it as BOSS 0’ (recess opacification). A similar discrepancy was also noted in the DSA interpretation, highlighting the intrinsic subjectivity in BOSS-based grading

Representative cases showing complete agreement between IVCBCT and DSA in the BOSS 1^33%^ and the BOSS 3 categories are shown in Figs. 1 and 2, respectively. These images support the ability of IVCBCT to accurately delineate minimal intra-device contrast opacification and contrast retention outside the WEB device as well as to clearly depict the device morphology, with a performance comparable to that of DSA. A representative case of inter-reader discrepancy is illustrated in Fig. 3. One reader classified the occlusion status as BOSS 0 (complete occlusion), whereas the other classified the same status—subtle contrast opacification within the proximal recess—as BOSS 0’. Interestingly, a similar discrepancy was also observed in DSA interpretation, underscoring the inherent subjectivity associated with BOSS-based assessment.

## Discussion

Our study demonstrated that IVCBCT is a reliable, non-invasive imaging modality for the follow-up of intracranial aneurysms treated with the WEB device, showing perfect agreement with DSA in aneurysm occlusion grading and high interobserver consistency. Notably, our study applied a refined 8‑point grading system based on the BOSS, including the subcategorization of intra-device filling (BOSS 1^33%^, 1^66%^, and 1^100%^). Despite this increased granularity, both intermodality (IVCBCT vs. DSA) and interobserver agreement remained exceptionally high (κ = 1.0 and 0.86, respectively), highlighting the robustness and reproducibility of IVCBCT in the detailed evaluation of WEB-treated aneurysms.

MRI-based techniques, including TOF-MRA and contrast enhanced-MRA, have limitations in the detection of residual aneurysms due to magnetic susceptibility artifacts caused by the radiopaque markers of the WEB device [12]. Timsit et al. reported low concordance between MRA and DSA (κ = 0.36) and suboptimal interobserver agreement [13]. Although ultra-short echo time (UTE)-MRI has been proposed to improve visualization by reducing susceptibility effects [13, 14], Toth et al. demonstrated that despite improved visualization of the WEB device and adjacent vessels using contrast-enhanced UTE-MRI, recanalization within the device could not be evaluated [15]. This is likely due to the intrinsic magnetic radiofrequency shielding effect created by the dense nitinol and platinum mesh of the WEB device, especially at the proximal and distal markers. These components cause local field inhomogeneity and rapid T2* dephasing, resulting in a signal dropout that obscures slow or stagnant flow within the device.

CTA provides better anatomical details than MRA. Raoult et al. reported that CTA shows good agreement with DSA (κ = 0.61 for occlusion, κ = 1.0 for compression), outperforming TOF-MRA [16]. However, similar to MRI, CTA suffers from artifacts that limit the assessment of intra-device flow.

Compared with these modalities, IVCBCT demonstrated superior diagnostic performance and reproducibility in our study. Its ability to provide high-resolution, artifact-reduced images of both the device and adjacent vessels enables accurate assessment of the aneurysm occlusion status. Complete concordance with DSA results supports its utility as a reliable follow-up tool post-WEB implantation. IVCBCT may reduce the need for invasive DSA procedures, potentially minimizing patient risk and healthcare costs. Its integration into routine follow-up protocols could enhance the safety and efficiency of posttreatment monitoring in patients with intracranial aneurysms treated with the WEB device. Although the cost and operational feasibility of IVCBCT may vary across healthcare systems, it is noteworthy that in countries such as Japan, where angiography systems are widely available and imaging reimbursement is tightly regulated, IVCBCT can be cheaper than contrast-enhanced CT or MRI. Therefore, the clinical applicability of IVCBCT should be interpreted in light of local infrastructure and economic context, since these factors are likely to shape the integration of IVCBCT into standard follow-up procedures. Importantly, in our study, interobserver disagreement occurred in only two cases, both involving the differentiation between BOSS 0 and BOSS 0’. This minor discrepancy might be attributed to mild artifacts caused by the proximal radiopaque marker of the WEB device, which can obscure the fine visualization of the aneurysm neck on IVCBCT. As shown in Fig. 3, a minor difference in interpretation arose from subtle contrast retention within the recess, which was graded as BOSS 0 by one reader and as BOSS 0’ by the other. This underscores the need for careful standardization when assessing borderline occlusion grades. Nevertheless, because both BOSS 0 and BOSS 0’ are classified as adequate occlusions, this variation does not have any clinical consequences or impact on patient management.

Our findings also align with those of prior studies investigating the clinical significance of persistent intra-device contrast opacification, commonly referred to as BOSS 1 or the “contrast-in-WEB” phenomenon [10]. Caroff et al. reported this finding in 9.1% of WEB-treated aneurysms on cone-beam CT and identified associations with dual antiplatelet therapy, low aspect ratio, and the use of WEB-17 [10]. Importantly, these cases were not associated with aneurysm rupture or retreatment. Similarly, Nguyen et al. found contrast-in-WEB in 6.3% of their cohort, with no rupture observed during follow-up and only one case requiring retreatment due to dome opacification [17]. These studies suggest that BOSS 1 may frequently represent a benign imaging finding. However, the long-term clinical significance of persistent contrast within the WEB device remains unclear; these findings may represent stable contrast retention or the early stages of recanalization. In this context, IVCBCT offers a critical advantage: the ability to noninvasively and accurately visualize the entire device lumen and its interface with the aneurysm wall in three dimensions. This high level of detail can help detect subtle morphological changes that may precede clinical recurrence and guide follow-up intensity and treatment decisions. Given the diagnostic uncertainty surrounding BOSS 1, our findings underscore the value of IVCBCT as a sensitive and reproducible tool for the monitoring of WEB-treated aneurysms.

### Limitations

This study had several limitations. First, it was conducted at a single institution with a relatively small sample size of 16 patients, which may limit the generalizability of the findings to a broader population. The study exclusively included patients with unruptured wide-neck intracranial aneurysms treated with the WEB device alone. Consequently, these results may not apply to patients with ruptured aneurysms, those treated with adjunctive devices, or those with aneurysms of different morphologies. Further validation with larger multicenter cohorts and extended follow-up periods is needed to confirm its diagnostic performance and clinical utility across diverse patient populations.

Additionally, although IVCBCT offers high spatial resolution and reduced metal artifact interference, it is a static imaging modality that lacks temporal resolution. This limitation prevents the assessment of dynamic contrast flow, which may be useful for distinguishing between stable contrast retention and early signs of recanalization. In this regard, DSA remains superior for evaluating real-time hemodynamic behaviors.

## Conclusions

IVCBCT offers a non-invasive, accurate, and reproducible method for assessing aneurysm occlusion post-WEB treatment, demonstrating complete concordance with DSA. Its adoption could reduce the reliance on invasive procedures, enhance patient safety, and lower healthcare costs. These findings support the integration of IVCBCT into routine follow-up protocols for patients treated using the WEB devices.

## Data Availability

The datasets generated and analyzed during the current study are available from the corresponding author upon reasonable request.

## References

[1] Pierot L, Gubucz I, Buhk JH, et al. Safety and efficacy of aneurysm treatment with the WEB: results of the WEBCAST 2 study. ajnr am J Neuroradiol. 2017;38:1151–5. 10.3174/ajnr.A5178. 28450432 10.3174/ajnr.A5178 PMC7960101

[2] Hendricks BK, Yoon JS, Yaeger K, et al. Wide-neck aneurysms: systematic review of the neurosurgical literature with a focus on definition and clinical implications. J Neurosurg. 2020;133:159–65. 10.3171/2019.3.JNS183160. 31200376 10.3171/2019.3.JNS183160

[3] Pierot L, Costalat V, Moret J, et al. Safety and efficacy of aneurysm treatment with WEB: results of the WEBCAST study. J Neurosurg. 2016;124:1250–6. 10.3171/2015.2.JNS142634. 26381253 10.3171/2015.2.JNS142634

[4] Pierot L, Moret J, Turjman F, et al. WEB treatment of Intracranial aneurysms: clinical and anatomical results in the french observatory. AJNR Am J Neuroradiol. 2016;37:655–9. 10.3174/ajnr.A4578. 26514608 10.3174/ajnr.A4578 PMC7960156

[5] Nam HH, Jang DK, Cho BR. Complications and risk factors after digital subtraction angiography: 1-year single-center study. J Cerebrovasc Endovasc Neurosurg. 2022;24:335–40. 10.7461/jcen.2022.E2022.05.001. 36153862 10.7461/jcen.2022.E2022.05.001 PMC9829562

[6] Buhk JH, Lingor P, Knauth M. Angiographic CT with intravenous administration of contrast medium is a noninvasive option for follow-up after intracranial stenting. Neuroradiology. 2008;50:349–54. 10.1007/s00234-007-0342-x. 18246336 10.1007/s00234-007-0342-x PMC2275774

[7] Gölitz P, Struffert T, Knossalla F, et al. Angiographic CT with intravenous contrast injection compared with conventional rotational angiography in the diagnostic work-up of cerebral aneurysms. AJNR Am J Neuroradiol. 2012;33:982–7. 10.3174/ajnr.A2883. 22268091 10.3174/ajnr.A2883 PMC7968818

[8] Feldkamp LA, Davis LC, Kress JW. Practical cone-beam algorithm. J Opt Soc Am A. 1984;1:612–9. 10.1364/JOSAA.1.000612.

[9] Caroff J, Mihalea C, Tuilier T, et al. Occlusion assessment of intracranial aneurysms treated with the WEB device. Neuroradiology. 2016;58:887–91. 10.1007/s00234-016-1715-9. 27312475 10.1007/s00234-016-1715-9

[10] Caroff J, Mihalea C, Dargazanli C, et al. Persistent opacification of the woven endobridge device: a conebeam CT analysis of the bicêtre occlusion scale score 1 phenomenon. AJNR Am J Neuroradiol. 2023;44:291–6. 10.3174/ajnr.A7783. 36759143 10.3174/ajnr.A7783 PMC10187822

[11] Landis JR, Koch GG. The measurement of observer agreement for categorical data. Biometrics. 1977;33:159–74. 10.2307/2529310. 843571

[12] Timsit M, Soize S, Chassin O, et al. Diagnostic accuracy of 3D time-of-flight and contrast-enhanced MR angiography at 3T compared with digital subtraction angiography for the evaluation of aneurysm occlusion after woven endobridge treatment. AJNR Am J Neuroradiol. 2016;37:1684–9. 10.3174/ajnr.A4791. 27102311 10.3174/ajnr.A4791 PMC7984701

[13] Irie R, Suzuki M, al Yamamoto Met. Assessing blood flow in an intracranial stent: a feasibility study of silent MR angiography. AJNR Am J Neuroradiol. 2015;36:967–70. 10.3174/ajnr.A4213. 25523588 10.3174/ajnr.A4199 PMC7990590

[14] Ayabe Y, Hamamoto K, Yoshino Y, et al. Ultra-short echo-time MR angiography combined with a subtraction method to assess intracranial aneurysms treated with a flow-diverter device. Magn Reson Med Sci. 2023;22:117–25. 10.2463/mrms.tn.2021-0106. 34897149 10.2463/mrms.tn.2021-0106 PMC9849415

[15] Toth D, Sommer S, Ludovichetti R, et al. Visualization of intracranial aneurysms treated with woven endobridge (WEB) devices using ultrashort echo time magnetic resonance imaging (UTE-MRI). AJNR Am J Neuroradiol. 2025;46:107–12. 10.3174/ajnr.A8042. 38997121 10.3174/ajnr.A8401 PMC11735423

[16] Raoult H, Eugène F, Le Bras A, et al. CT angiography for one-year follow-up of intracranial aneurysms treated with the WEB device: utility in evaluating aneurysm occlusion and WEB compression at one year. J Neuroradiol. 2018;45:343–8. 10.1016/j.neurad.2018.02.010. 29524499 10.1016/j.neurad.2018.02.010

[17] Nguyen HA, Soize S, Manceau PF, et al. Persistent blood flow inside the woven endobridge device more than 6 months after Intracranial aneurysm treatment: frequency, mechanisms, and management—A retrospective single-center study. AJNR Am J Neuroradiol. 2020;41:1225–31. 10.3174/ajnr.A6593. 32527839 10.3174/ajnr.A6593 PMC7357643

